# “An increase in COVID-19 patients would be overwhelming”: A qualitative description of healthcare workers’ experiences during the first wave of COVID-19 (March 2020 to October 2020) at Malawi’s largest referral hospital.

**DOI:** 10.12688/wellcomeopenres.17368.1

**Published:** 2022-02-04

**Authors:** Felix Limbani, Blessings M Kapumba, Henry Mzinganjira, Tamara Phiri, Henry C Mwandumba, Jamie Rylance, Ben Morton, Nicola Desmond

**Affiliations:** 1 Malawi Liverpool Wellcome Trust Clinical Research Programme, Kamuzu University of Health Sciences, PO Box 30096, Chichiri, Blantyre, BT3, Malawi; 2 Queen Elizabeth Central Hospital, Blantyre, BT3, Malawi; 3 Liverpool School of Tropical Medicine, Liverpool, L3 5QA, UK

**Keywords:** COVID-19, Health care workers, perspectives, Low-income country, Queen Elizabeth Central Hospital

## Abstract

Background

COVID-19 is currently a global health threat. Healthcare workers are on the front-line of the COVID-19 outbreak response and therefore at heightened risk of infection. There is a dearth of evidence from Sub-Saharan Africa about healthcare worker experiences in managing COVID-19.  We have reported on healthcare worker responses, experiences, and perspectives on epidemic response strategies at Queen Elizabeth Central Hospital, Malawi’s largest referral hospital.

Methods

We conducted 39 face-to-face in-depth interviews with a purposively selected sample of healthcare workers during the first wave of COVID-19 in Malawi (March 2020 to October 2020). The study included healthcare workers who provided direct and indirect patient care.

Results

During the early phase of the first wave (March to May 2020), healthcare workers expressed concerns with inadequate working space, unconducive infrastructure, delayed and rushed training on the management of COVID-19, and lack of incentives. Additionally, the hospital had staff shortages and limited essential resources such as piped oxygen and personal protective equipment. This increased healthcare worker fears of contracting COVID-19 and they were less willing to volunteer at COVID-19 isolation units. Resource constraints and limited preparedness compromised the care pathway particularly with increased numbers of COVID-19 patients. By the peak of the first wave (June to August 2020) many of these issues had been resolved. The hospital provided refresher training courses, personal protective equipment became available, incentives were offered to healthcare workers working in COVID-19 units and piped oxygen was installed. Staff morale was boosted, and more staff were willing to work at the COVID-19 isolation centres.

Conclusion

Experiences of healthcare workers during the first wave of COVID-19 are critical for improving care in future COVID-19 waves. Response strategies in resource-constrained areas should prioritise timely training of staff, creation of adequate isolation areas, provision of adequate medical supplies and strengthening leadership.

## Background

More than a year into the pandemic, COVID-19 remains a health concern worldwide with over 200 million confirmed cases and more than four million deaths. There is now cause for optimism as over six billion doses of the COVID-19 vaccine have been administered globally ^1^. Malawi, a low-income country in Southern Africa, registered 61,800 cases and 2,302 deaths, as of the 1 ^st^ November 2021 ^2^. After Malawi’s first confirmed case of COVID-19 (2 ^nd^ April 2020), Blantyre became the epicenter of the national epidemic. In response, Queen Elizabeth Central Hospital (QECH), the largest referral hospital in Malawi based in Blantyre, was reorganised to respond to this need ^3^. As COVID-19 cases spread throughout the country, the need for informed context-specific initial experiences of healthcare workers (HCWs) became essential.

Healthcare workers (HCWs) are at the frontline of the COVID-19 outbreak response and at increased risk of infection ^4^. Risks include exposure to pathogens, long working hours, psychological distress, fatigue, stigma, and potential for physical and psychological violence, both in and outside the hospital setting ^4^. Literature from other low- and middle-income countries on HCW experience of managing COVID-19 reported heavy workloads, insufficient personal protective equipment (PPE), and the fear amongst HCWs of becoming infected or infecting others ^5, 6^. At the start of the COVID-19 pandemic in Malawi, health workers in major hospitals in Malawi protested over limited availability of PPE, shortages in the healthcare workforce, lack of training on COVID-19 case management and the lack of a “risk allowance” for HCWs caring for COVID-19 patients ^7^.

The World Health Organization (WHO) developed a series of protocols to guide HCWs in infection prevention and control (IPC) for COVID-19. Recommendations for clinical management of COVID-19 cases include the establishment of effective patient-flow with proper screening, triaging, and targeted referral ^8^. However, in the event of an epidemic, additional measures such as reverse triage * ^1^ may be required to create surge capacity within the healthcare system ^9^. Healthcare workers are required to make difficult decisions on how to ration health resources ^10^. Guidelines developed in high-income countries may not be feasible in low resource settings.

There is limited literature from sub-Saharan Africa on HCW experience with management of COVID-19. Most studies have taken place in high income settings and primarily through quantitative surveys ^5, 6^. Our aim was to explore HCW responses, experiences, and perspectives on epidemic response strategies in a low-income and resource-constrained health system. Understanding HCW thoughts and perspectives toward Queen Elizabeth Central Hospital’s (QECH) COVID-19 response strategies is key to strengthening health systems and improving patient care.

## Methods

### Study design

We conducted a hospital-based qualitative descriptive study with a purposively selected sample of HCWs using one-to-one interviews. Gathering perspectives of individual HCWs that directly experienced the first wave of COVID-19 at the hospital ensured representation of a range of views that balanced individual depth with understanding a broad range of perspectives ^11^. Views of HCWs presented in this manuscript are reported from the first wave of COVID-19 in Malawi (March 2020 to October 2020).

### Study site and population

The study was conducted at QECH, Blantyre, Malawi’s largest government referral hospital. At the time of the study, the hospital had a bed capacity of 1000. Sixty beds were available for COVID-19 patients. The hospital directly serves a population of 800,000 (city of Blantyre) and is the tertiary referral centre for 7.75 million (Southern Region) ^12^. It is one of four central hospitals in the country. Participants in the study were HCWs involved with both direct and indirect patient care ( Table 1). This range of cadres ensured perspectives of both decision-makers and frontline workers were included. Between 20 ^th^ October and 13 ^th^ November 2020, a total of 39 eligible HCWs (18 males and 21 females) were enrolled and interviewed. Data saturation was used to determine the sample size (we stopped collecting data when no new ideas were emerging from the interviews). Potential participants were identified by QECH departmental managers and introduced to the research team. We then gave them study information and five days to decide on their involvement.

**Table 1.** Cadres of HCWs interviewed.

HCWs	Sex		Total
	Male	Female	
Management staff	1	2	3
Consultants	1	1	2
Junior doctors	2	2	4
Clinical officers	3	1	4
Nurses	5	5	10
Auxiliary nurses		4	4
Laboratory technicians	2	1	3
Patient attendants	1	2	3
Hospital attendants	2	2	4
Receptionist/ data clerk	1	1	2
**Total**	**18**	**21**	**39**

**Figure 1.**
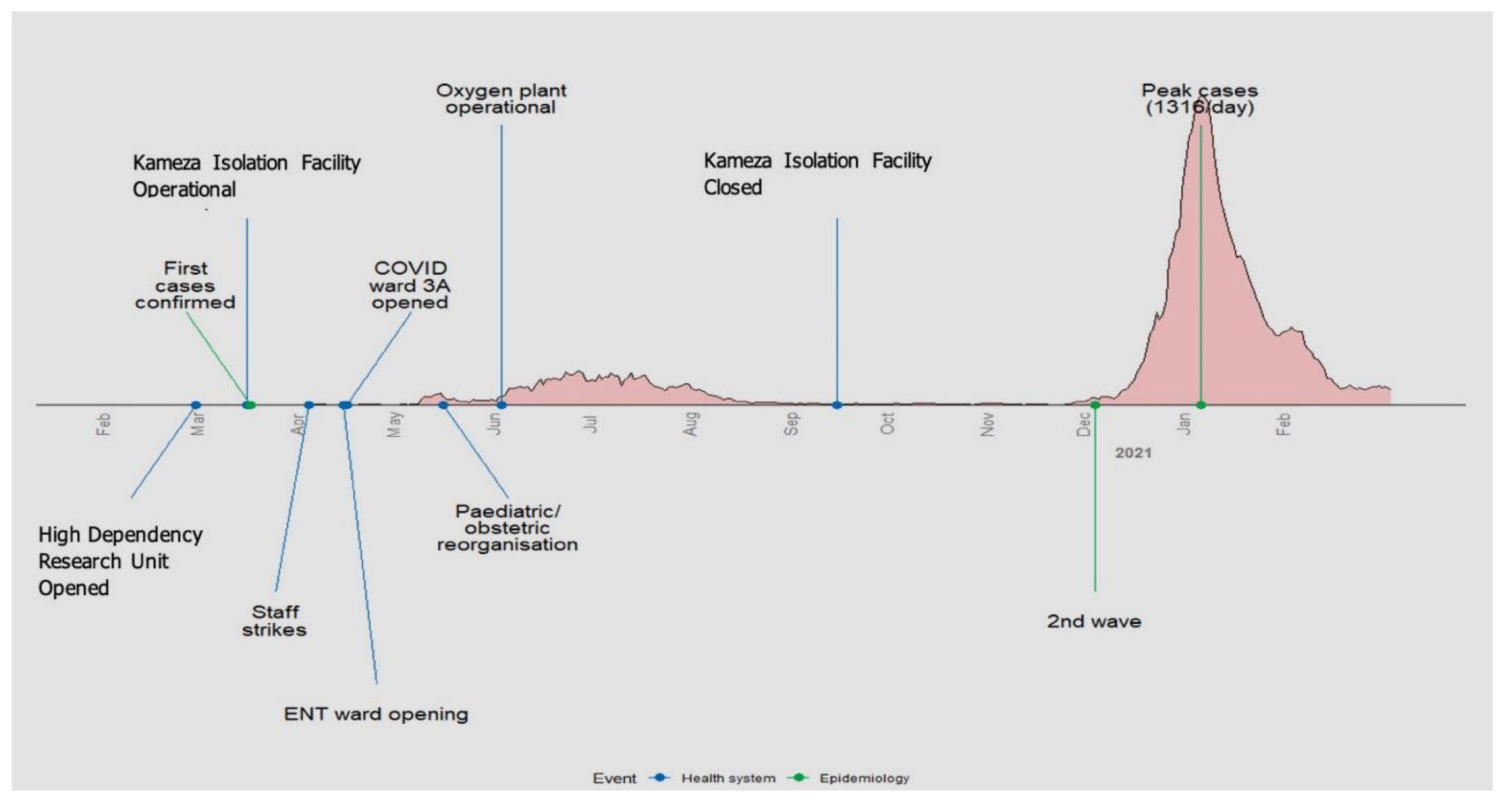
Timeline of events.

Malawi’s healthcare system comprises primary level facilities (community-based health services), secondary level facilities (district hospitals) and tertiary level facilities (regional central hospitals) ^13^. The levels are linked by referral systems. The healthcare system has a large burden of infectious diseases such as HIV/AIDS, tuberculosis and malaria ^14^. The system is also affected by critical shortage of healthcare providers (doctors, nurses and midwives) ^15^. Initially, a COVID-19 isolation centre was developed separate from QECH, and under the management of the District Health Office (DHO) ( Figure 1). The capacity of the DHO isolation centre was limited which led to creation of isolation units within QECH ^16^.

### Data collection

All interviews were conducted within the hospital setting, at a time and place convenient for participants to minimize disturbing their workflow. We explored participant experiences and priorities for COVID-19 epidemic response strategies in the hospital and adaption of international protocols. These included their concerns, as well as recommendations for responding to increased cases of COVID-19. Interviews were conducted either in English or Chichewa (local dialect) by FL and BK. All interviews were audio-recorded using a Victure V3 digital voice recorder, transcribed verbatim into written Chichewa or English (depending on language used), and translated into English where necessary. Following each interview, audio files and transcripts were saved on a secure MLW network drive, which was only accessible to the study team. Participant data was de-identified in the transcripts including file names; instead, identification numbers were used. Participants were financially compensated for their time according to Malawi’s published guidance on research participant compensation ^17^.

### Data analysis

We used thematic content analysis. All transcribed and translated data were transferred to the qualitative data analysis software package (NVIVO 12 - QSR International, Warrington, UK). Two researchers (FL and BK) undertook initial coding of a small number of transcripts and agreed on a codebook for further coding. The data was initially coded deductively from the study objectives and inductively using sub-themes emerging from the data. It was analysed following broad descriptive themes across individuals. A cyclical and iterative approach to analysis was followed to identify emerging themes that were then clarified or explored through further individual interviews. To ensure that the results from the interviews were trustworthy, we discussed them with a small group of selected participants. Both researchers, FL with background in health systems research and BK with a clinical background, have considerable understanding of health systems in Southern Africa and experience in qualitative research.

## Results

**Figure 2.**
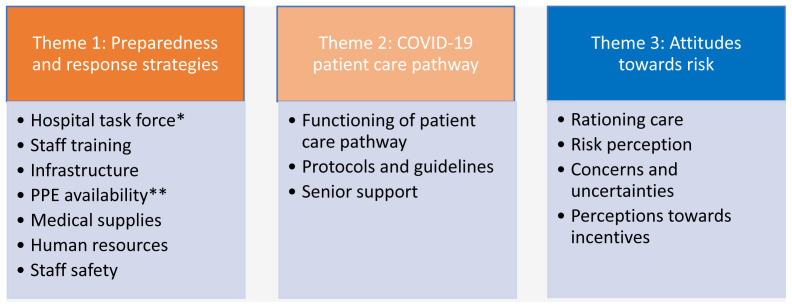
Presentation of thematic areas that inductively and deductively emerged from the data. *Task force denotes a special team that led COVID-19 response in the hospital. **PPE denotes Personal Protective Equipment.

We present our findings under three key themes: 1) perspectives on preparedness and response strategies; 2) descriptions of the COVID-19 patient care pathway; and 3) attitudes towards risk. Figure 2.

### Views on COVID-19 preparedness and response strategies

The hospital employed a multidisciplinary approach when preparing and coordinating risk-mitigation activities. Participants pointed to examples such as staff training, establishment of infrastructure, personal protective equipment, drugs and medical supplies, human resources, and staff safety, protection, and support.

***The hospital COVID-19 task force.*** A special response team (“taskforce”) worked in four sub teams: patient and community education, hygiene (infection control), clinical care, and administration. The taskforce was responsible for coordination of all COVID-19 related activities including designing and setting up of care facilities, organising staff training, and putting measures to observe patients and staff safety.

While the taskforce impacted on good practice, not all HCWs were aware of its existence, its members, or leadership:

“ *No, I don’t know. I will just assume there was one, because whenever there is something new, every hospital sets up a task force. So, I assume there should be one. I am just not aware who the head is.” ( **Consultant)***
*“There is a team that made the protocols, made sure that the PPEs were available, the team that trained the nurses. Doctors, nurses and the administration staff were part of the team, but I don’t know who they were.”* ( ***Nurse)***

Amongst those who were aware of the task force (mostly those in leadership positions), there was a positive perception of preparedness for managing COVID-19 cases at the hospital, highlighting as examples staff training and establishment of isolation facilities. While the task force was comprised of different frontline HCW cadres, there were also some members of management involved, creating a link between decision-makers and frontline workers.

***Staff training.*** All workers at the hospital underwent COVID-19 training. For some participants, the training helped to address fears that existed among HCWs and prepared them to face the pandemic. They felt that the training imparted the required knowledge and expertise on IPC, management of COVID-19 patients, use of equipment, and contact tracing ( Table 2). Refresher training, on the job training, peer support and continuous professional development (CPD) sessions, helped to further improve HCW expertise. 

**Table 2.** COVID-19 clinical management training topics compiled from QECH COVID-19 training materials.

Topic number	Description
**Topic 1**	The state of COVID pandemic. Malawi and QECH preparedness
**Topic 2**	Screening and Triaging of COVID-19 suspects
**Topic 3**	WHO Basic Emergency Care: approach to the acutely ill and injured
**Topic 4**	COVID-19 disease evaluation and management
**Topic 5**	Nursing evaluation and management of a COVID-19 case
**Topic 6**	COVID-19 specimen collection and handling
**Topic 7**	Monitoring a COVID-19 patient in care
**Topic 8**	Discharge and follow-up of COVID-19 cases
**Topic 9**	Psychological First Aid for COVID-19 patients
**Topic 10**	Mental and psychological support for HCWs
**Topic 11**	Infection prevention measures. Recommendations for clinical care delivery
**Topic 12**	Coordination and support: referral, consultation, and supervision
**Topic 13**	COVID-19 pathway walk-through

*“I remember one day we were told how to adjust oxygen, whether to increase or decrease. It was a certain doctor who came and called some of us who were on duty and explained to us how it is done. Then pasting the guidelines on the walls.” ( **Nurse)***

While training of HCWs was strongly recommended, the majority across all cadres felt that the process of organising the training was delayed and that it was delivered in a hasty manner. Having some staff attend the training affected ward coverage by staff despite efforts to deliver the training in phases. Some senior HCWs felt the delay in conducting the training was due to lack of funding as they depended on external funders.

*“I understand it was a crisis but maybe we could have been more organised, like scheduling them in good time. As nurses or clinicians, we have rotas so if you just say tomorrow there’s three days training, it was really a headache to release people and then to find others to work on their behalf. There was chaos sort of.” ( **Nurse)***

In relation to the training package and content, some doctors wished it was separated among cadres, to avoid loading HCWs with unnecessary information.

*“For the content of training it was yes relevant, but I still think that they should have divided at some points to say this is only for clinicians, and this is just for the nursing team. There are things that one cadre has to learn more about and then the other ones can’t actually do.” ( **Junior doctor)***

**Figure 3.**
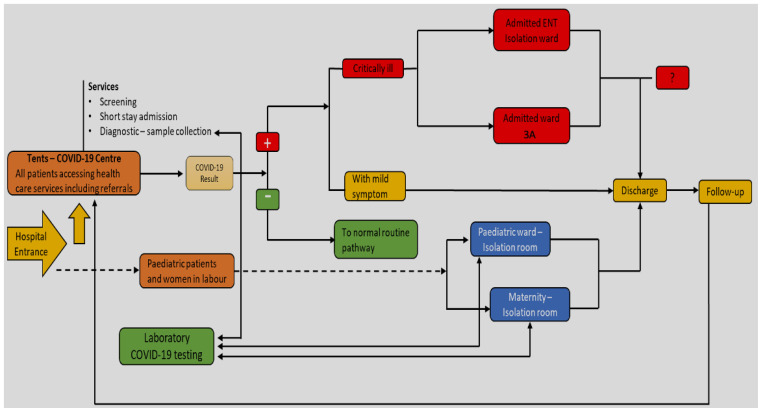
Clinical pathway of patients during COVID-19.

**Figure 4.**
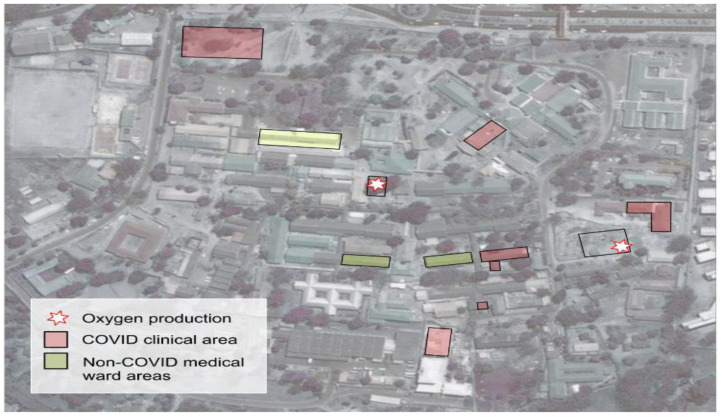
Aerial view of hospital infrastructure.

***Establishment of infrastructure.*** Most HCWs said that the hospital (QECH) prepared to respond to COVID-19 by ensuring additional space was created such as erecting tents at the main gate for screening, sample collection, and cohorting suspected cases of COVID-19 until test results were confirmed. In addition, the ENT * ^2^ unit and ward 3A * ^3^ and High Dependency Respiratory Unit (HDRU) were turned into isolation centres for patients requiring admission. Ward 1A * ^4^ was reserved for pregnant women diagnosed with COVID-19. Clinical pathways of patients and centres where COVID-19 activities were taking place is illustrated in Figure 3 and Figure 4 respectively.

Whilst the majority of participants recognised this as a good initiative, some felt that the quality and size of the structures were inadequate for the increasing number of COVID-19 cases.

*“Ward 3A is 36 bed and the HDRU has about 6-8 beds. ENT has more space. So, we would take close to around 50 patients. We also have 1A for obstetric patients. So, if it is beyond 60, it would be chaotic for the hospital.” ( **Nurse)***

In certain instances, the hospital’s overall infrastructure setup did not meet guideline recommended IPC requirements. Some participants expressed concerns with poor ventilation in some wards, inadequate space that resulted in doffing and donning of PPE in the same room; use of the same toilet facilities by HCWs and patients; and use of the same sluice by wards with and without COVID-19 patients.

Most HCWs, particularly those who worked at the hospital's main gate (tents), thought that the tents constructed at the entrance were undesirable for performing some medical procedures or accommodating patients during extreme weather conditions.

*“The buildings are very poor, and I don’t know what will happen if rains have started. Maybe these things (tents) will fall off. Some three weeks ago, there were some rains, it was terrible. It was so dirty here. I was on night shift and there was mud everywhere and even where we sleep.” ( **Junior doctor)***

Some participants commended the initial plans to treat patients in a pre-exiting isolation facility constructed in response to Ebola outbreaks in the region ^16^. Building separate infrastructure away from hospital was preferred as an ideal alternative, although this was not done. 

***Personal protective equipment.*** Whilst the majority of the participants stated that resources for IPC such as PPE, were made available when staff needed them, others expressed frustration with the lack of PPE.

*“Provision of PPE to be honest, was frustrating. On paper we were told that we have PPE, but access was difficult. They will tell you that every ward has PPE but when you go to the ward, you don’t have it.” **(Junior doctor)***

Heads of departments oversaw the distribution and monitoring of PPE to clinicians, while unit matrons oversaw nurses and other HCW cadres. PPE included gloves, hazmat suits, gowns, goggles, surgical masks, N95 masks, gumboots and scrubs. HCWs, on the other hand, felt that PPE was mostly supported by external partners.

*“For the affiliates [partners that work along with QECH for clinical support and research] to be honest they did a good job, like they offered tents, they offered PPE, offered teaching but as far as the ministry is concerned, I felt a bit let down. I felt like we didn’t prepare.” ( **Junior doctor)***

There were two groups of participants who indicated that PPE was not equally available and accessible in the hospital. HCWs working in other wards felt that appropriate PPE was prioritised for COVID-19 wards only, while in other wards they only had surgical masks and gowns. Secondly, lower cadres such as hospital attendants, patient attendants, and auxiliary nurses felt that nurses and doctors were the only HCWs with sufficient PPE. The lower cadres reported that they had to search for PPE in the morning and use it for the whole day while attending to different patients.

*“Sometimes we would carry the patients without enough protective clothing like the gowns. You would find that the gowns are finished, because people have scrambled for them in the morning. To find another one it was difficult and now there is a new patient to attend to.” ( **Hospital attendant)***

In contrast, clinical staff perceived unnecessary use of PPE among lower cadres. All staff including attendants engaged in screening at the hospital gate wanted to use extensive PPE, leading to shortages elsewhere. Clinical staff perceived that fear that existed among HCWs resulted in inappropriate use of PPE.

*“Another shortfall is to know when to use PPE. For example, health surveillance assistants who are there [screening gate], you may find they are putting on the PPEs from head to toe, which is not necessary, but those are special for procedures, maybe when you want to handle COVID death that’s when you use that, but simply you can just put on a mask.” ( **Nurse)***

***Drugs and medical supplies.*** There were mixed responses regarding availability of medication intended for COVID-19 management. These included: heparin (unfractionated and enoxaparin), anti-hypertensives, insulin, and oxygen. Some HCWs reported intermittent stock outs during this period but that these were quickly rectified. However, there was frustration with only short courses of medication being issued by the hospital pharmacy.

*“But if I go once more [to the pharmacy], I will not be provided with sufficient medication, I will still be given few medications which means I had to go the following day since the dosage which was prescribed for the patient was for three days and the patient had only taken medication for only a single day, so I will go back with notes that I came yesterday.” ( **Hospital attendant)***

Participants also mentioned that there were no reported stock issues for drugs commonly used in the management of critical COVID-19 patients such as dexamethasone and ceftriaxone.

*“The good thing with drugs fund is that they give us a three-month funding, and during this period it means you readily have the monies. And when they say the drugs are out, we could quickly process the requisition and buy the drugs.” ( **Administrative staff)***

***Human resources.*** All respondents expressed concerns over underlying limited human resource capacity, particularly when they had to attend to both COVID-19 and other patients. Participants described staffing as a chronic problem not only at the hospital but nationally. COVID-19 was perceived to aggravate this situation with diversion of resources from already understaffed areas required to build COVID-19 surge capacity. HCWs raised concern of becoming overwhelmed and burnt out.

*“We have about four nurses working and sometimes they get sort of overwhelmed. So, if we were able to have more people working on the ground, I think that would be really helpful.” ( **Consultant)***
*“Our schedules had to adjust because we had to cover our normal wards as well as COVID patients. That was incorporated on our rotas depending on where you were, you would either have to do your normal ward and then you had to see the COVID patients as well.” ( **Junior doctor)***

Pre-existing HCW shortages were further strained when some HCWs from all cadres were diagnosed with COVID-19 and required to isolate. Nursing students were withdrawn from the wards by their schools early in the pandemic and fear of contracting COVID-19 reduced the number of personnel further.

***Staff safety, protection, and support.*** During the early phases of the pandemic, safety concerns led to protests and strikes ^7^ ( Figure 1). The hospital addressed the issue through training and production of education materials; limiting visitor access to hospital premises; and placing hand washing facilities, water, soap, and sanitisers around the hospital.

*“People were genuinely concerned about their safety. There was misunderstanding about the required PPE and personal safety. Willingness to work was at a minimum and that’s what led to the sit-in that the ministry intervened.” ( **Consultant)***

HCWs initiated their own strategies to protect each other including reminding, checking and correcting each other on appropriate and optimal IPC approaches.

*“Before entering a COVID-19 ward, a friend has to check you if you have dressed properly. But we had a problem with our doctors because most of them want to do things in a hurry without observing IPC procedures. So, our duty was to say, please don’t go in unless you do this and that.” ( **Nurse)***

Although staff with comorbidities such as diabetes mellitus were supposed to be working in non-COVID clinical areas, it was perceived that this was only partially successful.

*“Although they informed us that COVID-19 was mostly affecting those with diabetes, BP and asthma, the hospital was not able to protect HCWs with those illnesses. They said people with such diseases will be transferred to other departments where there is less patient contact, but that did not happen.” ( **Hospital attendant)***

HCWs in lower cadres felt excluded from information pertaining to patient COVID-19 status.

“ *I think we are not really valued; they only warn each other at the top when there is a threat. You will find that someone is sending you right there without even cautioning you [that there is a COVID patient] but some would whisper to you, not as a group but whisper to you.” **(Hospital attendant)***

### COVID-19 patient care pathways

Figure 3 describes four possible ways that COVID-19 patients accessed the hospital; were screened; and then subsequently tested and treated. Screening was done at the hospital main gate. For patients suspected to have COVID-19, samples were taken for testing at the QECH laboratory with results available in 24 hours. Patients were held in tents as they waited for test results and received treatment for their immediate symptoms. Of patients that tested positive for COVID-19, those with mild or moderate symptoms were discharged and could go home, while those with severe symptoms were admitted to COVID-19 wards.

The maternity unit had a designated COVID-19 room in which all pregnant women in labour were admitted upon arrival at the hospital. Screening and testing of pregnant women were done whilst obstetric care was provided. Patients that tested negative were transferred to the normal maternity ward. Those that tested positive were transferred to a COVID-19 isolation ward within the department for COVID-19 obstetric patients. Paediatric patients had a separate care pathway created within paediatric unit. Patients attending outpatient clinics were screened at the main gate. Further screening was also done in the departments where the clinics were being held.

***Functioning of patient care pathways.*** We explored HCW perception of how well the patient care pathway functioned at QECH. HCWs felt that the introduction of screening at the main gate and closure of other entry gates was important.

*“This is a hospital where there are a lot of people just hanging around without knowing what they are doing. The hospital limited numbers in the ward areas, making sure that everyone that is coming in has been screened. The hospital did well on that one.” ( **Nurse)***

However, there was concern about limited screening at the hospital entrance gate during out of hours and congestion in screening areas during morning and lunch hours; laboratory result delays; lack of effective isolation of COVID-19 patients within the wider hospital; mental distress for patients who witnessed other COVID-19 patients dying; poor medical equipment; and shortage of HCWs. Additional concerns included limited access to piped oxygen in the waiting areas and oxygen cylinders being the only source of oxygen in some wards. This was a concern particularly for patients requiring high flow oxygen where the cylinder would run out quickly.

*“We were losing some of the patients at the tents because we were not prepared enough. Whilst a patient waiting for test results needs high flow (piped) oxygen, and it’s not there because the patient is using oxygen cylinder and yet he or she needs a lot of oxygen.” ( **Clinical officer)***

Whilst managing the patients, HCWs perceived that patients that had comorbidities such as asthma and hypertension were particularly affected by COVID-19. HCWs had confidence in the treatments they were able to offer and felt these benefited their patients.

*“We needed to find out what comorbidities did one have. Then we were able to stage them. People with asthma and coronary disease, oxygenation was important. When we give them oxygen early and some medication like dexamethasone and heparin, we were helping them a lot.” ( **Nurse)***

***Protocols and guidelines for managing COVID-19 patients.*** Most doctors confirmed that they had access to guidelines and protocols for COVID-19 management. They found that training was useful to encourage systematised care. However, fewer nurses and clinical officers were aware of management protocols and felt they required guidance from senior staff.

*“They are not available. We can’t find them that these are the protocols. If I want, I will talk to a consultant who will help me like, ok this is how we do it. From my experience I can tell you the time I have been helping the COVID patients, I have never seen a guideline.” **(Nurse)***

Senior clinicians had adapted existing treatment protocols from multiple sources including WHO case management guidelines to suit the local context.

*“So, in our local context for QECH it (adapting guidelines) worked. Other countries were ventilating patients. But we said we are not doing that basing on the fact that 1: we only have 4 ventilators the hospital is quite big, it caters for the whole of the southern region so if we were to say we will be ventilating all COVID patients it means we would fail in managing other cases.” ( **Junior doctor)***

***Senior support.*** HCWs, especially in lower cadres, agreed that senior clinical support was available to manage COVID-19 patients. Support was provided by both senior nurses and doctors who provided bedside training

*“After the trainings, we now have CPD sessions with nurses during morning handovers. The sessions are at departmental level and are very effective because they are more interactive. That is how we get updated on what it is we are supposed to do.” ( **Doctor)***

There was also senior support around staff welfare and risk management. HCWs in COVID-19 wards were offered dedicated transport to and from work and a dedicated quarantine centre after completion of their block of shifts.

*“I made a decision that I should not be home because I have my siblings and parents. So, I talked with top management, and they told me that there is a place I could be staying at College of Medicine, so I stayed there.” ( **Nurse)***

However, gaps in supporting HCWs existed. HCWs said that not all issues presented to management were addressed. Multiple HCWs experienced fear, anxiety, and emotional distress. Despite the presence of mental health services in the hospital, there was a lack of formal support for HCWs who experienced these issues.

*“People were only discussing that maybe people should try be seen by psychologist or psychiatrist, but nothing formal was actually communicated so I wouldn’t really say that the hospital had implemented this to cover the psychological aspect of the healthcare workers.” **(Junior doctor)***

### Attitudes towards risk and rationing of care

***Attitude and perceptions on rationing of care.*** HCWs were concerned that the hospital would be overwhelmed with COVID-19 admissions. Rationing care would be affected by limited access to oxygen cylinders; shortage of human resources; inadequate space; and burnout for those involved in direct patient care. Some respondents felt that non-COVID-19 wards would suffer because resources were being prioritised for COVID-19 wards.

*“We have 20 places for oxygen and if we are to receive 30 patients then the rest will suffer because we will then have to remove the oxygen from patients who have reached 92 (blood oxygen saturation level) and then put the one with 85, so the moment you are removing from this patient to give it to another patient then this one will be dropping so we would have lots of people dying.” **(Nurse)***

Most HCWs perceived that patients of higher social status should not be prioritised when providing care. They felt that it was ethically incorrect to treat patients differently based on their social status but rather that they should prioritise those that are likely to recover. Others felt that patients of higher social status should not be prioritised despite having the potential to influence improvements in the health system.

HCWs had mixed views toward the concept of reverse triage ^9^. While some felt this was a possible method to reduce hospital congestion, others felt it could increase deaths as patients would be discharged in critical state. Reverse triage was already happening in the hospital. While HCWs were screening and discharging those that were healthier, some HCWs said that they had seen a lot of patients leaving the hospital that looked unwell.

*“Reverse triage works. If a patient is able to take drugs orally, is fit enough to walk without any sort of discomfort that patient should go home. But patient satisfaction reduces if you reverse triage. People comply to go home but within a week they are back, so eventually you see the same patients over and over again frequenting the hospital.” **(Consultant)***

***Risk perception.*** HCWs overwhelmingly felt at high risk of COVID-19 infection. HCWs were particularly fearful of putting their families at risk. They were anxious that many people including HCWs were dying in countries with better resourced health systems. Factors that heightened risk perception included: perceived restrictions to PPE supply and instruction on usage (staff preferred higher grade PPE, even in low clinical risk areas); uncertainty around optimal COVID-19 management; working in poorly ventilated areas; and fellow HCWs testing positive for COVID-19.

*“The risk is very high, more especially for us (ENT department). We look at the nose and the throat on a daily basis, that’s the dwelling place of COVID-19. If they happen to cough while you are examining the throat, you are at a risk.” ( **Clinical officer)***

HCWs minimised their risk by reducing exposure time, abiding by infection prevention procedures. Some also used alternative remedies including taking ginger and steam inhalation. 

***Concerns and uncertainties.*** HCWs were worried about a decline in IPC implementation amongst fellow HCWs. HCWs felt that the government should have invested in dedicated isolation centres. They were also concerned about loss of support to their families should they get the virus and die. HCWs in COVID-19 wards were concerned that some of the problems they had raised with management were not being addressed.

*“We proposed structural changes up to now they haven’t been sorted out. We had issues with communication, we didn’t have a phone. That was addressed but we still have airtime challenges. We have communicated with management time and again, it hasn’t been sorted up to now.” ( **Nurse)***

HCWs, particularly those in the lower cadres, wanted more information on the risk of contracting COVID-19 from cadavers compared to patients, the cause of relapse in the number of cases later in the wave, why COVID-19 was more common in men, whether they would be reinfected after recovering from COVID-19 and how the virus gets cleared from the body when viruses such as HIV cannot be cured.

*“I hear COVID gets cleared from the body. My understanding about viruses, - once you are infected, they will stay with you for the rest of your life. Once infected with HIV, you will always be HIV positive. So, how does COVID-19 gets cleared in the end?” ( **Laboratory technician)***

***Perceptions towards incentives.*** There was a strong sense of inequity and resentment around the provision of additional monetary allowances for HCWs. This was a major factor in the staff walkouts at the beginning of the crisis with HCWs unhappy that senior managers and officials were allegedly given allowances with no provision for lower cadres. Allowances were made available for staff working in COVID-19 cohort wards, but these were not extended to non-COVID-19 areas. This was a continuing source of resentment amongst staff.

*“The circular indicated that all staff working in COVID wards must receive allowance based on his/her grade and indeed we started receiving. In winter, things changed, we were told that we were no longer entitled to such allowances. They said this because we won’t touch the patients yet when these patients are coming from the tent, the first person they meet is me because I have to take all their details. We presented our concerns to all the bosses and we have given up.” ( **Data clerk)***

Most HCWs agreed that monetary allowances did not remove risk but were an important token to demonstrate appreciation. Some preferred to term this as motivation allowance rather than risk allowance. Participants viewed the allowance as support for the additional costs incurred by working on COVID-19 duties.

*“I will give you an example of people who clean in the COVID ward, their risk is the same as doctor. Sometimes maybe more than us, but the sort of compensation they get as in their salary is quite low. When COVID came even fares for mini-buses went up but these people were expected every day to come to work. They don’t have cars like maybe doctors.” ( **Junior doctor)***

## Discussion

HCW perspectives on preparation and management of COVID-19 patients demonstrate that they had to contend with a number of issues during the early phase of the first wave. There was a lack of resources including shortages of staff and equipment such as piped oxygen and unequally distributed PPE. Limited preparation resulted in delayed training, inadequate working space, unconducive infrastructure, and resentment over incentives and limited leadership response to staff safety concerns. Unallayed fears of COVID-19 infection affected HCWs’ willingness to volunteer in COVID-19 care, promoted inappropriate use of PPE and use of alternative prevention remedies. Lack of communication, compounded with lack of information sharing, left some HCWs unsure of what was expected of them. However, the hospital worked hard to cope with the first wave of COVID-19. By the peak of the first wave, many of these issues had been resolved. The hospital provided refresher training; PPE was made available; incentives were offered; piped oxygen was installed; and more staff were willing to work at the COVID-19 isolation centres.

Reports of deaths from COVID-19 among HCWs in high-income countries with better-resourced health systems instilled fear among HCWs at the hospital who were aware of the many resource constraints. HCWs feared that an increase in the number of COVID-19 patients at QECH would be overwhelming. Lack of adequate space and resources meant that HCWs would be at greater risk. Being exposed to such an environment was likely to increase their personal risk of contracting COVID-19 and, in turn, expose their families. This fear among HCWs was aggravated by the behaviour of other staff who stopped abiding by infection prevention and control procedures because the numbers of COVID-19 cases started declining.

Demand for staff incentives such as risk allowances and additional benefits delayed the response initiatives. Baseline salaries for HCWs in Malawi are low such that additional allowances are a major motivational tool. Many employees also provide financial support to their extended families. HCWs were thus unsure of their own welfare and that of their families in the event they became infected and died which further affected their willingness to work.

The first COVID-19 wave in Malawi was fortunately slow with relatively few COVID-19 admissions in comparison to high-income countries. Although the admissions were low, the issues identified had the potential to hamper an effective response. The hospital response to COVID-19 described in this study highlights the impact an epidemic has on under-resourced healthcare services. Coping strategies in such constrained settings such as the suspension of chronic disease services are also likely to contribute to poor patient outcomes. 

Despite the above challenges, the hospital's COVID-19 response ensured that COVID-19 patient management was guided by international protocols adapted to local context, and that senior support was available at all levels of the care pathway. Open and proactive leadership, with no disconnect between those in management and frontline providers, is ideal in addressing system challenges in the epidemic response. In the case of QECH, addressing structural concerns raised by HCWs; protecting high-risk staff; sharing information with lower cadres; and engaging staff on appropriate use of PPE would have instilled increased HCW’s confidence.

In many LMICs, there is a shortage of personal protective equipment; inadequate protocols for use; insufficient staff (exacerbated by self-isolation); limited space and conversion of other hospital infrastructure into COVID-19 isolation centres ^6, 18– 20^; a lack of stable and reliable oxygen supply; and increased levels of stress among HCWs as a result of fear of infection and infecting others ^5, 6^. Our study findings report similar experiences. A survey of hospitals in Malawi identified several of these contextual challenges, including limited isolation rooms; personal protective equipment; and access to oxygen in medical wards ^21^. Fragile health systems in Africa are susceptible to severe outbreaks ^22^.

Our findings are consistent with those from high-income countries, which have seen a growing disparity between supply and demand for medical resources, including hospital bed shortages; personal protective equipment shortages; and staff shortages ^10, 23^. During the COVID-19 pandemic, recommendations for equitable resource allocation included maximizing benefits; treating people equally; promoting and rewarding instrumental value (prioritizing those who can save others); and prioritizing those from lower-economic backgrounds ^10^. However, in addition to rationing care, other issues raised by HCWs, such as prioritizing COVID-19 wards with PPE, and delaying training due to a lack of funds (mostly covering allowances); impose ethical constraints; and necessitate context-specific recommendations.

This study had several strengths. The study interviewed various cadres of secondary healthcare workers, including those who were directly involved in the management of COVID-19 patients and those who were not, ensuring a representative sample of perspectives. Both interviewers were Malawians with extensive knowledge of the Malawian health system and previous experience conducting qualitative research in the hospital. We conducted this study near the conclusion of the first wave, allowing HCWs to reflect on how the wave began, peaked, and slowed. However, we captured perspectives from a single health facility which cannot fully represent the heterogeneity of resources, COVID rates and wider situations of other hospitals. We cannot exclude the potential for bias in recruitment or question responses as a result of the participant compensation, but the study approach is in line with all others of its type in Malawi.

## Conclusion

HCWs have been at the forefront of the fight against COVID-19, and their role has contributed to people’s care and support during epidemics. HCWs demonstrated tenacity in managing patients despite the difficulties inherent in providing care at COVID-19 isolation centres.

Our findings highlight a variety of concerns and issues to consider when developing epidemic response strategies including timely training of staff, creating enough and conducive space for patient isolation, and having adequate resources. In addition, our findings suggest that hospitals should have a well-defined patient care pathway with clearly defined processes, inputs, and outputs, and are continuously monitored by the response committee.

Other areas for improvement include providing mental healthcare support for HCWs on the frontline; providing appropriate compensation to HCWs; engaging patients and communities; and ensuring that HCWs adhere to IPC guidelines.

## Ethics

The study was granted ethics clearance from the College of Medicine Research Ethics Committee - COMREC (certificate number P.08/20/3098) and Liverpool School of Tropical Medicine Research Ethics Committee - LSTM (certificate number 20-059). All participants provided written consent, agreed to have their deidentified data shared and were made aware that despite anonymization, qualitative data can inadvertently reveal the identity of participants. All participants were offered nominal monetary tokens of appreciation according to Malawi’s published guidance on research participant compensation. We have provided a reflexivity statement on our research partnership in line with recent consensus recommendations ^24^.

## Data availability

### Underlying data

Figshare: An increase in COVID-19 patients would be overwhelming” - Voices of Healthcare workers from Malawi’s largest referral hospital during COVID-19 first wave. https://doi.org/10.6084/m9.figshare.17136161 ^25^

This project contains the following underlying data:

- Transcripts for in-depth interviews with healthcare workers

Data are available under the terms of the Creative Commons Attribution 4.0 International license (CC-BY 4.0).
