# Supplementary material for: “An increase in COVID-19 patients would be overwhelming”: A qualitative description of healthcare workers’ experiences during the first wave of COVID-19 (March 2020 to October 2020) at Malawi’s largest referral hospital
Source: Wellcome Open Res. 2022 Feb 4;7:40. [Version 1] doi: 10.12688/wellcomeopenres.17368.1 (PMC10036953; doi:10.12688/wellcomeopenres.17368.1)
Supplement: Peer review history

# Peer review: Reviewer response for version 1

**DOI:** 10.21956/wellcomeopenres.19203.r55440

**Published:** 2023-03-23

**Authors:** Aya Mostafa

**Affiliations:** 1 Department of Community, Environmental and Occupational Medicine, Faculty of Medicine, Ain Shams University, Cairo, Cairo Governorate, Egypt

Thank you for the opportunity to review the manuscript. The article is one of the few in Africa that qualitatively explored HCWs experiences during the COVID-19 pandemic. The results are rich and could inform are more in-depth discussion. Here are some comments/questions to improve the quality and added value of the manuscript:

  

- Why was the “purposive” sampling technique selected for this study? What type of purposive sampling was done and on what basis? 

- The authors mention “a total of 39 eligible HCWs”. What were the eligibility criteria? How many HCWs were excluded based on these criteria?

- What was the advantage of using in-depth one-to-one interviews over focus group discussions in this study? What are the possible disadvantages of lacking focus group discussions as a qualitative method in this study? Please address this in the discussion/limitations section.

- Is the term “descriptive” an appropriate description of the type of qualitative study used here? It may not be applicable in this study because the authors analyze the interviews and derive themes thereof.

- Could the authors provide the study tools (interview guide) as supplementary material?

- Why did the authors highlight in particular the phrase: ““An increase in COVID-19 patients would be overwhelming” in the title, among all statements provided by HCWs?

- Were all interviews conducted after the training took place? It would be good for the reader to know, as this may greatly affect participants’ responses. Most quoted responses communicate a negative overall picture of COVID-19 response by the management. The evidence of the following statement could not be found in the manuscript: “By the peak of the first wave (June to August 2020) many of these issues had been resolved.”, as the authors mention in the abstract. For instance, there are no quotes supporting that “incentives were offered to healthcare workers working in COVID-19 units”, as claimed in the abstract. If there were interviews before and after these improvements took place, it would be good to indicate which and how many and cite quotes from those as well.

- The discussion somehow is not benefiting from the qualitative nature of this study and is written as if the results were quantitative. The qualitative perspective of why the shortages happened and how the process could be improved are not sufficiently addressed. This part is of importance for pinning down the added value and usefulness for other similar resource-limited settings.

Is the work clearly and accurately presented and does it cite the current literature?

Yes

If applicable, is the statistical analysis and its interpretation appropriate?

Yes

Are all the source data underlying the results available to ensure full reproducibility?

Partly

Is the study design appropriate and is the work technically sound?

Yes

Are the conclusions drawn adequately supported by the results?

Partly

Are sufficient details of methods and analysis provided to allow replication by others?

Partly

Reviewer Expertise:

Public health and Epidemiology

I confirm that I have read this submission and believe that I have an appropriate level of expertise to confirm that it is of an acceptable scientific standard.

# Peer review: Reviewer response for version 1

**DOI:** 10.21956/wellcomeopenres.19203.r51108

**Published:** 2022-10-24

**Authors:** Matthew F. Chersich

**Affiliations:** 1 Wits Reproductive Health and HIV Institute, Faculty of Health Sciences, University of the Witwatersrand, Johannesburg, South Africa

The paper covers a critical topic and applies robust methods and solid analysis, a very pleasing read. My concern is about the timing of this publication in terms of the phase of the pandemic. The lag from research concept to publication is a major problem that I share! I wonder if the discussion can mention that a bit more... How much is relevant for future lessons, or is learning from an experience and knowledge gained sufficient? The findings are very interesting, the rapid improvements in the workplace are remarkable, showing what is possible in a resource-constrained setting with adequate attention.

- One highly relevant topic that was not examined is vaccine hesitancy among health workers. It is a major problem in South Africa, with vaccine refusal rates as high as 25%. Figure 1 is very useful in documenting this important series of events.
- It would be interesting to give a sense of how many healthcare workers were involved in COVID care at the facility.
- Some more information on what the training entailed would be useful: duration of the training and facilitator type, for example.
- I would be interested to know more about whether the interventions done were top-down, dictated by the MoH and the task force, or bottom-up where health workers and others identified solutions based on their lived experiences. The text on the tents and mud is perhaps an example of a solution prescribed without consultation. Were decisions made hastily, compromising the quality of them?
- More might be said about the comparison between the Ebola and COVID response.
- Can anything be said about any long-lasting benefits of the changes made, behavioral, infrastructure, clinical experience, or team culture?
- The discussion could provide more reflection on potential solutions to the problems raised in the results. After reading such a rich results section, I was left a bit disappointed by the discussion. What are potential solutions to the incentives issue? Some info about how these problems were tackled in other African settings might be useful in the discussion.

Is the work clearly and accurately presented and does it cite the current literature?

Yes

If applicable, is the statistical analysis and its interpretation appropriate?

Yes

Are all the source data underlying the results available to ensure full reproducibility?

Partly

Is the study design appropriate and is the work technically sound?

Yes

Are the conclusions drawn adequately supported by the results?

Yes

Are sufficient details of methods and analysis provided to allow replication by others?

Yes

Reviewer Expertise:

Climate change

I confirm that I have read this submission and believe that I have an appropriate level of expertise to confirm that it is of an acceptable scientific standard.
